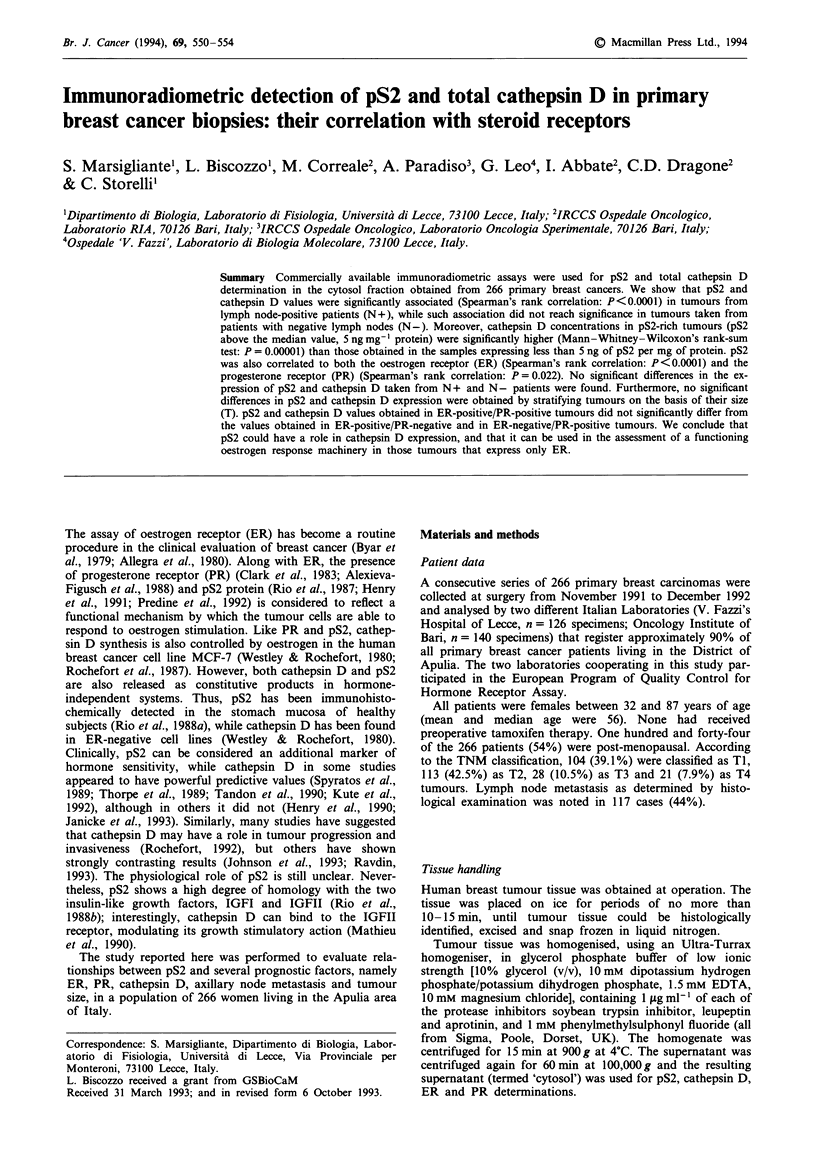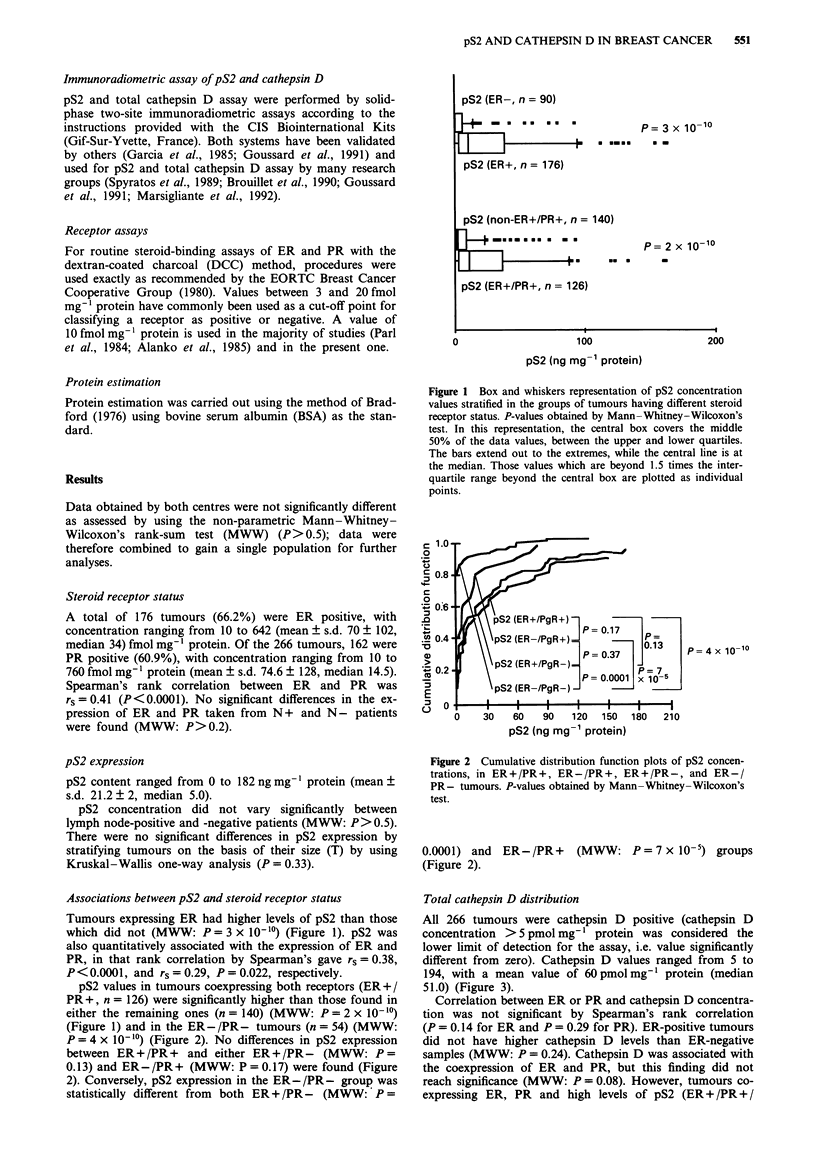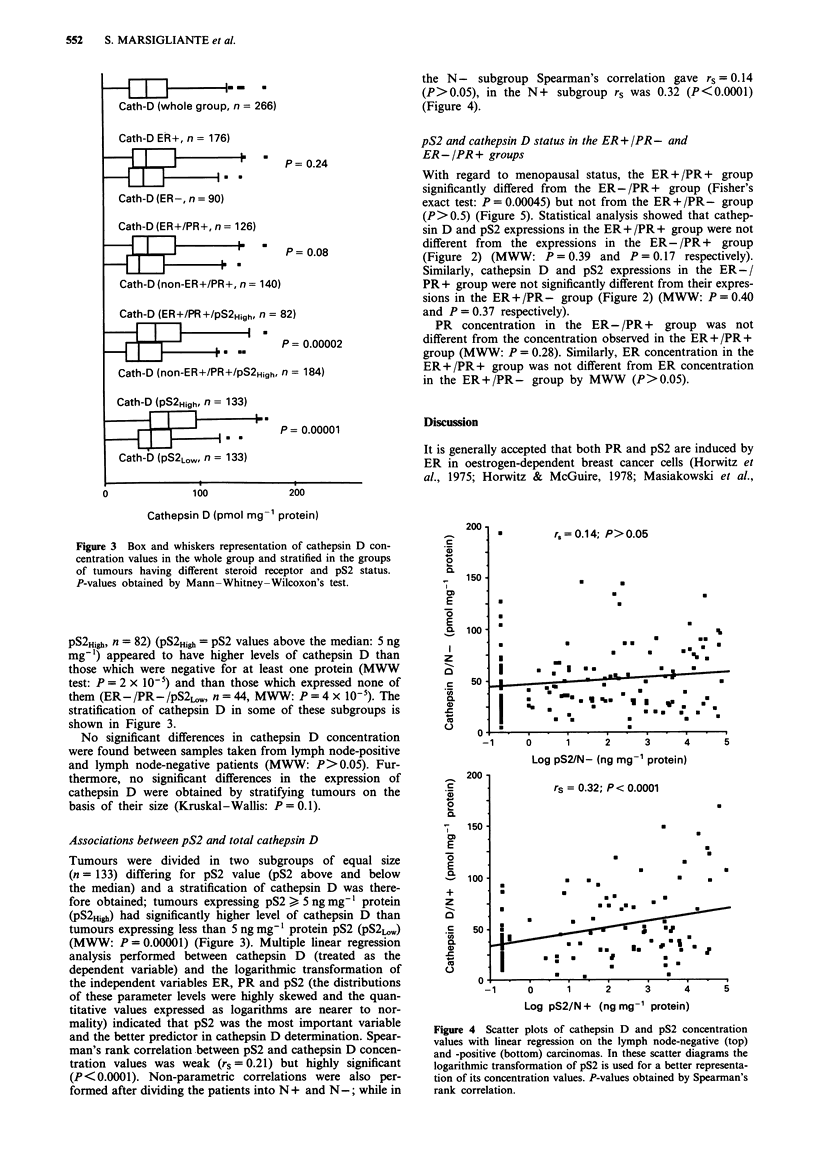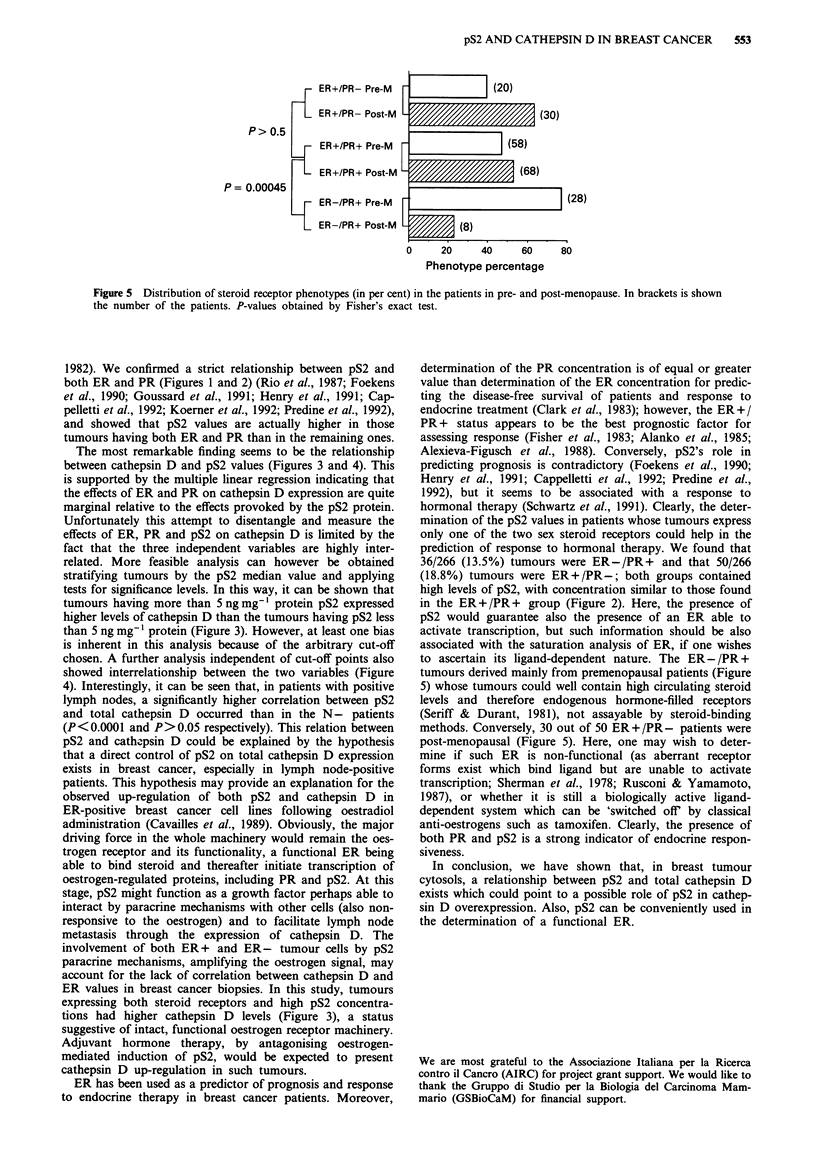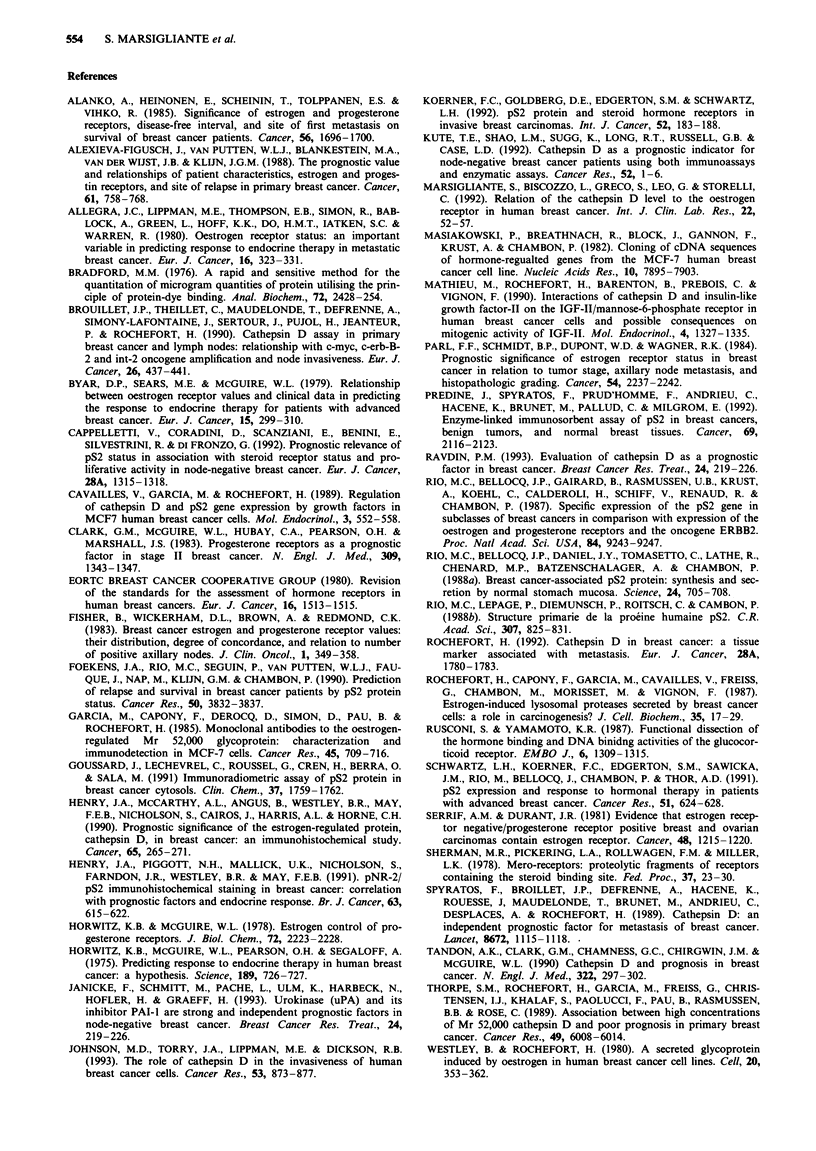# Immunoradiometric detection of pS2 and total cathepsin D in primary breast cancer biopsies: their correlation with steroid receptors.

**DOI:** 10.1038/bjc.1994.100

**Published:** 1994-03

**Authors:** S. Marsigliante, L. Biscozzo, M. Correale, A. Paradiso, G. Leo, I. Abbate, C. D. Dragone, C. Storelli

**Affiliations:** Dipartimento di Biologia, Università di Lecce, Italy.

## Abstract

Commercially available immunoradiometric assays were used for pS2 and total cathepsin D determination in the cytosol fraction obtained from 266 primary breast cancers. We show that pS2 and cathepsin D values were significantly associated (Spearman's rank correlation: P < 0.0001) in tumours from lymph node-positive patients (N+), while such association did not reach significance in tumours taken from patients with negative lymph nodes (N-). Moreover, cathepsin D concentrations in pS2-rich tumours (pS2 above the median value, 5 ng mg-1 protein) were significantly higher (Mann-Whitney-Wilcoxon's rank-sum test: P = 0.00001) than those obtained in the samples expressing less than 5 ng of pS2 per mg of protein. pS2 was also correlated to both the oestrogen receptor (ER) (Spearman's rank correlation: P < 0.0001) and the progesterone receptor (PR) (Spearman's rank correlation: P = 0.022). No significant differences in the expression of pS2 and cathepsin D taken from N+ and N- patients were found. Furthermore, no significant differences in pS2 and cathepsin D expression were obtained by stratifying tumours on the basis of their size (T). pS2 and cathepsin D values obtained in ER-positive/PR-positive tumours did not significantly differ from the values obtained in ER-positive/PR-negative and in ER-negative/PR-positive tumours. We conclude that pS2 could have a role in cathepsin D expression, and that it can be used in the assessment of a functioning oestrogen response machinery in those tumours that express only ER.


Br. J. Cancer (1994), 69, 550–554 © Macmillan Press Ltd., 1994

### Immunoradiometric detection of pS2 and total cathepsin D in primary breast cancer biopsies: their correlation with steroid receptors

S. Marsigliante[1], L. Biscozzo[1], M. Correale[2], A. Paradiso[3], G. Leo[4], I. Abbate[2], C.D. Dragone[2] & C. Storelli[1]

[1]*Dipartimento di Biologia, Laboratorio di Fisiologia, Università di Lecce, 73100 Lecce, Italy;* [2]*IRCCS Ospedale Oncologico, Laboratorio RIA, 70126 Bari, Italy;* [3]*IRCCS Ospedale Oncologico, Laboratorio Oncologia Sperimentale, 70126 Bari, Italy;* [4]*Ospedale 'V. Fazzi', Laboratorio di Biologia Molecolare, 73100 Lecce, Italy.*

**Summary** Commercially available immunoradiometric assays were used for pS2 and total cathepsin D determination in the cytosol fraction obtained from 266 primary breast cancers. We show that pS2 and cathepsin D values were significantly associated (Spearman's rank correlation: $P < 0.0001$) in tumours from lymph node-positive patients (N+), while such association did not reach significance in tumours taken from patients with negative lymph nodes (N−). Moreover, cathepsin D concentrations in pS2-rich tumours (pS2 above the median value, 5 ng mg$^{-1}$ protein) were significantly higher (Mann–Whitney–Wilcoxon's rank-sum test: $P = 0.00001$) than those obtained in the samples expressing less than 5 ng of pS2 per mg of protein. pS2 was also correlated to both the oestrogen receptor (ER) (Spearman's rank correlation: $P < 0.0001$) and the progesterone receptor (PR) (Spearman's rank correlation: $P = 0.022$). No significant differences in the expression of pS2 and cathepsin D taken from N+ and N− patients were found. Furthermore, no significant differences in pS2 and cathepsin D expression were obtained by stratifying tumours on the basis of their size (T). pS2 and cathepsin D values obtained in ER-positive/PR-positive tumours did not significantly differ from the values obtained in ER-positive/PR-negative and in ER-negative/PR-positive tumours. We conclude that pS2 could have a role in cathepsin D expression, and that it can be used in the assessment of a functioning oestrogen response machinery in those tumours that express only ER.

The assay of oestrogen receptor (ER) has become a routine procedure in the clinical evaluation of breast cancer (Byar *et al.*, 1979; Allegra *et al.*, 1980). Along with ER, the presence of progesterone receptor (PR) (Clark *et al.*, 1983; Alexieva-Figusch *et al.*, 1988) and pS2 protein (Rio *et al.*, 1987; Henry *et al.*, 1991; Predine *et al.*, 1992) is considered to reflect a functional mechanism by which the tumour cells are able to respond to oestrogen stimulation. Like PR and pS2, cathepsin D synthesis is also controlled by oestrogen in the human breast cancer cell line MCF-7 (Westley & Rochefort, 1980; Rochefort *et al.*, 1987). However, both cathepsin D and pS2 are also released as constitutive products in hormone-independent systems. Thus, pS2 has been immunohistochemically detected in the stomach mucosa of healthy subjects (Rio *et al.*, 1988*a*), while cathepsin D has been found in ER-negative cell lines (Westley & Rochefort, 1980). Clinically, pS2 can be considered an additional marker of hormone sensitivity, while cathepsin D in some studies appeared to have powerful predictive values (Spyratos *et al.*, 1989; Thorpe *et al.*, 1989; Tandon *et al.*, 1990; Kute *et al.*, 1992), although in others it did not (Henry *et al.*, 1990; Janicke *et al.*, 1993). Similarly, many studies have suggested that cathepsin D may have a role in tumour progression and invasiveness (Rochefort, 1992), but others have shown strongly contrasting results (Johnson *et al.*, 1993; Ravdin, 1993). The physiological role of pS2 is still unclear. Nevertheless, pS2 shows a high degree of homology with the two insulin-like growth factors, IGFI and IGFII (Rio *et al.*, 1988*b*); interestingly, cathepsin D can bind to the IGFII receptor, modulating its growth stimulatory action (Mathieu *et al.*, 1990).

The study reported here was performed to evaluate relationships between pS2 and several prognostic factors, namely ER, PR, cathepsin D, axillary node metastasis and tumour size, in a population of 266 women living in the Apulia area of Italy.

Correspondence: S. Marsigliante, Dipartimento di Biologia, Laboratorio di Fisiologia, Università di Lecce, Via Provinciale per Monteroni, 73100 Lecce, Italy.
L. Biscozzo received a grant from GSBioCaM
Received 31 March 1993; and in revised form 6 October 1993.

### Materials and methods

### *Patient data*

A consecutive series of 266 primary breast carcinomas were collected at surgery from November 1991 to December 1992 and analysed by two different Italian Laboratories (V. Fazzi's Hospital of Lecce, $n = 126$ specimens; Oncology Institute of Bari, $n = 140$ specimens) that register approximately 90% of all primary breast cancer patients living in the District of Apulia. The two laboratories cooperating in this study participated in the European Program of Quality Control for Hormone Receptor Assay.

All patients were females between 32 and 87 years of age (mean and median age were 56). None had received preoperative tamoxifen therapy. One hundred and forty-four of the 266 patients (54%) were post-menopausal. According to the TNM classification, 104 (39.1%) were classified as T1, 113 (42.5%) as T2, 28 (10.5%) as T3 and 21 (7.9%) as T4 tumours. Lymph node metastasis as determined by histological examination was noted in 117 cases (44%).

### *Tissue handling*

Human breast tumour tissue was obtained at operation. The tissue was placed on ice for periods of no more than 10–15 min, until tumour tissue could be histologically identified, excised and snap frozen in liquid nitrogen.

Tumour tissue was homogenised, using an Ultra-Turrax homogeniser, in glycerol phosphate buffer of low ionic strength [10% glycerol (v/v), 10 mM dipotassium hydrogen phosphate/potassium dihydrogen phosphate, 1.5 mM EDTA, 10 mM magnesium chloride], containing 1 $\mu$g ml$^{-1}$ of each of the protease inhibitors soybean trypsin inhibitor, leupeptin and aprotinin, and 1 mM phenylmethylsulphonyl fluoride (all from Sigma, Poole, Dorset, UK). The homogenate was centrifuged for 15 min at 900 *g* at 4°C. The supernatant was centrifuged again for 60 min at 100,000 *g* and the resulting supernatant (termed 'cytosol') was used for pS2, cathepsin D, ER and PR determinations.

*Immunoradiometric assay of pS2 and cathepsin D*

pS2 and total cathepsin D assay were performed by solid-phase two-site immunoradiometric assays according to the instructions provided with the CIS Biointernational Kits (Gif-Sur-Yvette, France). Both systems have been validated by others (Garcia *et al.*, 1985; Goussard *et al.*, 1991) and used for pS2 and total cathepsin D assay by many research groups (Spyratos *et al.*, 1989; Brouillet *et al.*, 1990; Goussard *et al.*, 1991; Marsigliante *et al.*, 1992).

*Receptor assays*

For routine steroid-binding assays of ER and PR with the dextran-coated charcoal (DCC) method, procedures were used exactly as recommended by the EORTC Breast Cancer Cooperative Group (1980). Values between 3 and 20 fmol $mg^{-1}$ protein have commonly been used as a cut-off point for classifying a receptor as positive or negative. A value of 10 fmol $mg^{-1}$ protein is used in the majority of studies (Parl *et al.*, 1984; Alanko *et al.*, 1985) and in the present one.

*Protein estimation*

Protein estimation was carried out using the method of Bradford (1976) using bovine serum albumin (BSA) as the standard.

### Results

Data obtained by both centres were not significantly different as assessed by using the non-parametric Mann-Whitney-Wilcoxon's rank-sum test (MWW) ($P > 0.5$); data were therefore combined to gain a single population for further analyses.

*Steroid receptor status*

A total of 176 tumours (66.2%) were ER positive, with concentration ranging from 10 to 642 (mean ± s.d. 70 ± 102, median 34) fmol $mg^{-1}$ protein. Of the 266 tumours, 162 were PR positive (60.9%), with concentration ranging from 10 to 760 fmol $mg^{-1}$ protein (mean ± s.d. 74.6 ± 128, median 14.5). Spearman's rank correlation between ER and PR was $r_S = 0.41$ ($P < 0.0001$). No significant differences in the expression of ER and PR taken from N+ and N− patients were found (MWW: $P > 0.2$).

*pS2 expression*

pS2 content ranged from 0 to 182 ng $mg^{-1}$ protein (mean ± s.d. 21.2 ± 2, median 5.0).

pS2 concentration did not vary significantly between lymph node-positive and -negative patients (MWW: $P > 0.5$). There were no significant differences in pS2 expression by stratifying tumours on the basis of their size (T) by using Kruskal-Wallis one-way analysis ($P = 0.33$).

*Associations between pS2 and steroid receptor status*

Tumours expressing ER had higher levels of pS2 than those which did not (MWW: $P = 3 \times 10^{-10}$) (Figure 1). pS2 was also quantitatively associated with the expression of ER and PR, in that rank correlation by Spearman's gave $r_S = 0.38$, $P < 0.0001$, and $r_S = 0.29$, $P = 0.022$, respectively.

pS2 values in tumours coexpressing both receptors (ER+/PR+, $n = 126$) were significantly higher than those found in either the remaining ones ($n = 140$) (MWW: $P = 2 \times 10^{-10}$) (Figure 1) and in the ER−/PR− tumours ($n = 54$) (MWW: $P = 4 \times 10^{-10}$) (Figure 2). No differences in pS2 expression between ER+/PR+ and either ER+/PR− (MWW: $P =$ 0.13) and ER−/PR+ (MWW: P = 0.17) were found (Figure 2). Conversely, pS2 expression in the ER−/PR− group was statistically different from both ER+/PR− (MWW: $P =$

**Figure 1** Box and whiskers representation of pS2 concentration values stratified in the groups of tumours having different steroid receptor status. *P*-values obtained by Mann-Whitney-Wilcoxon's test. In this representation, the central box covers the middle 50% of the data values, between the upper and lower quartiles. The bars extend out to the extremes, while the central line is at the median. Those values which are beyond 1.5 times the inter-quartile range beyond the central box are plotted as individual points.

**Figure 2** Cumulative distribution function plots of pS2 concentrations, in ER+/PR+, ER−/PR+, ER+/PR−, and ER−/PR− tumours. *P*-values obtained by Mann-Whitney-Wilcoxon's test.

0.0001) and ER−/PR+ (MWW: $P = 7 \times 10^{-5}$) groups (Figure 2).

*Total cathepsin D distribution*

All 266 tumours were cathepsin D positive (cathepsin D concentration $> 5$ pmol $mg^{-1}$ protein was considered the lower limit of detection for the assay, i.e. value significantly different from zero). Cathepsin D values ranged from 5 to 194, with a mean value of 60 pmol $mg^{-1}$ protein (median 51.0) (Figure 3).

Correlation between ER or PR and cathepsin D concentration was not significant by Spearman's rank correlation ($P = 0.14$ for ER and $P = 0.29$ for PR). ER-positive tumours did not have higher cathepsin D levels than ER-negative samples (MWW: $P = 0.24$). Cathepsin D was associated with the coexpression of ER and PR, but this finding did not reach significance (MWW: $P = 0.08$). However, tumours co-expressing ER, PR and high levels of pS2 (ER+/PR+/

**Figure 3** Box and whiskers representation of cathepsin D concentration values in the whole group and stratified in the groups of tumours having different steroid receptor and pS2 status. *P*-values obtained by Mann–Whitney–Wilcoxon's test.

$pS2_{High}$, $n = 82$) ($pS2_{High}$ = pS2 values above the median: 5 ng $mg^{-1}$) appeared to have higher levels of cathepsin D than those which were negative for at least one protein (MWW test: $P = 2 \times 10^{-5}$) and than those which expressed none of them (ER−/PR−/$pS2_{Low}$, $n = 44$, MWW: $P = 4 \times 10^{-5}$). The stratification of cathepsin D in some of these subgroups is shown in Figure 3.

No significant differences in cathepsin D concentration were found between samples taken from lymph node-positive and lymph node-negative patients (MWW: $P > 0.05$). Furthermore, no significant differences in the expression of cathepsin D were obtained by stratifying tumours on the basis of their size (Kruskal–Wallis: $P = 0.1$).

### *Associations between pS2 and total cathepsin D*

Tumours were divided in two subgroups of equal size ($n = 133$) differing for pS2 value (pS2 above and below the median) and a stratification of cathepsin D was therefore obtained; tumours expressing pS2 $\geqslant 5$ ng $mg^{-1}$ protein ($pS2_{High}$) had significantly higher level of cathepsin D than tumours expressing less than 5 ng $mg^{-1}$ protein pS2 ($pS2_{Low}$) (MWW: $P = 0.00001$) (Figure 3). Multiple linear regression analysis performed between cathepsin D (treated as the dependent variable) and the logarithmic transformation of the independent variables ER, PR and pS2 (the distributions of these parameter levels were highly skewed and the quantitative values expressed as logarithms are nearer to normality) indicated that pS2 was the most important variable and the better predictor in cathepsin D determination. Spearman's rank correlation between pS2 and cathepsin D concentration values was weak ($r_S = 0.21$) but highly significant ($P < 0.0001$). Non-parametric correlations were also performed after dividing the patients into N+ and N−; while in the N− subgroup Spearman's correlation gave $r_S = 0.14$ ($P > 0.05$), in the N+ subgroup $r_S$ was 0.32 ($P < 0.0001$) (Figure 4).

### *pS2 and cathepsin D status in the ER+/PR− and ER−/PR+ groups*

With regard to menopausal status, the ER+/PR+ group significantly differed from the ER−/PR+ group (Fisher's exact test: $P = 0.00045$) but not from the ER+/PR− group ($P > 0.5$) (Figure 5). Statistical analysis showed that cathepsin D and pS2 expressions in the ER+/PR+ group were not different from the expressions in the ER−/PR+ group (Figure 2) (MWW: $P = 0.39$ and $P = 0.17$ respectively). Similarly, cathepsin D and pS2 expressions in the ER−/PR+ group were not significantly different from their expressions in the ER+/PR− group (Figure 2) (MWW: $P = 0.40$ and $P = 0.37$ respectively).

PR concentration in the ER−/PR+ group was not different from the concentration observed in the ER+/PR+ group (MWW: $P = 0.28$). Similarly, ER concentration in the ER+/PR+ group was not different from ER concentration in the ER+/PR− group by MWW ($P > 0.05$).

### Discussion

It is generally accepted that both PR and pS2 are induced by ER in oestrogen-dependent breast cancer cells (Horwitz *et al.*, 1975; Horwitz & McGuire, 1978; Masiakowski *et al.*,

**Figure 4** Scatter plots of cathepsin D and pS2 concentration values with linear regression on the lymph node-negative (top) and -positive (bottom) carcinomas. In these scatter diagrams the logarithmic transformation of pS2 is used for a better representation of its concentration values. *P*-values obtained by Spearman's rank correlation.

**Figure 5** Distribution of steroid receptor phenotypes (in per cent) in the patients in pre- and post-menopause. In brackets is shown the number of the patients. $P$-values obtained by Fisher's exact test.

1982). We confirmed a strict relationship between pS2 and both ER and PR (Figures 1 and 2) (Rio *et al.*, 1987; Foekens *et al.*, 1990; Goussard *et al.*, 1991; Henry *et al.*, 1991; Cappelletti *et al.*, 1992; Koerner *et al.*, 1992; Predine *et al.*, 1992), and showed that pS2 values are actually higher in those tumours having both ER and PR than in the remaining ones.

The most remarkable finding seems to be the relationship between cathepsin D and pS2 values (Figures 3 and 4). This is supported by the multiple linear regression indicating that the effects of ER and PR on cathepsin D expression are quite marginal relative to the effects provoked by the pS2 protein. Unfortunately this attempt to disentangle and measure the effects of ER, PR and pS2 on cathepsin D is limited by the fact that the three independent variables are highly interrelated. More feasible analysis can however be obtained stratifying tumours by the pS2 median value and applying tests for significance levels. In this way, it can be shown that tumours having more than 5 ng mg$^{-1}$ protein pS2 expressed higher levels of cathepsin D than the tumours having pS2 less than 5 ng mg$^{-1}$ protein (Figure 3). However, at least one bias is inherent in this analysis because of the arbitrary cut-off chosen. A further analysis independent of cut-off points also showed interrelationship between the two variables (Figure 4). Interestingly, it can be seen that, in patients with positive lymph nodes, a significantly higher correlation between pS2 and total cathepsin D occurred than in the N− patients ($P < 0.0001$ and $P > 0.05$ respectively). This relation between pS2 and cathepsin D could be explained by the hypothesis that a direct control of pS2 on total cathepsin D expression exists in breast cancer, especially in lymph node-positive patients. This hypothesis may provide an explanation for the observed up-regulation of both pS2 and cathepsin D in ER-positive breast cancer cell lines following oestradiol administration (Cavailles *et al.*, 1989). Obviously, the major driving force in the whole machinery would remain the oestrogen receptor and its functionality, a functional ER being able to bind steroid and thereafter initiate transcription of oestrogen-regulated proteins, including PR and pS2. At this stage, pS2 might function as a growth factor perhaps able to interact by paracrine mechanisms with other cells (also non-responsive to the oestrogen) and to facilitate lymph node metastasis through the expression of cathepsin D. The involvement of both ER+ and ER− tumour cells by pS2 paracrine mechanisms, amplifying the oestrogen signal, may account for the lack of correlation between cathepsin D and ER values in breast cancer biopsies. In this study, tumours expressing both steroid receptors and high pS2 concentrations had higher cathepsin D levels (Figure 3), a status suggestive of intact, functional oestrogen receptor machinery. Adjuvant hormone therapy, by antagonising oestrogen-mediated induction of pS2, would be expected to present cathepsin D up-regulation in such tumours.

ER has been used as a predictor of prognosis and response to endocrine therapy in breast cancer patients. Moreover, determination of the PR concentration is of equal or greater value than determination of the ER concentration for predicting the disease-free survival of patients and response to endocrine treatment (Clark *et al.*, 1983); however, the ER+/PR+ status appears to be the best prognostic factor for assessing response (Fisher *et al.*, 1983; Alanko *et al.*, 1985; Alexieva-Figusch *et al.*, 1988). Conversely, pS2's role in predicting prognosis is contradictory (Foekens *et al.*, 1990; Henry *et al.*, 1991; Cappelletti *et al.*, 1992; Predine *et al.*, 1992), but it seems to be associated with a response to hormonal therapy (Schwartz *et al.*, 1991). Clearly, the determination of the pS2 values in patients whose tumours express only one of the two sex steroid receptors could help in the prediction of response to hormonal therapy. We found that 36/266 (13.5%) tumours were ER−/PR+ and that 50/266 (18.8%) tumours were ER+/PR−; both groups contained high levels of pS2, with concentration similar to those found in the ER+/PR+ group (Figure 2). Here, the presence of pS2 would guarantee also the presence of an ER able to activate transcription, but such information should be also associated with the saturation analysis of ER, if one wishes to ascertain its ligand-dependent nature. The ER−/PR+ tumours derived mainly from premenopausal patients (Figure 5) whose tumours could well contain high circulating steroid levels and therefore endogenous hormone-filled receptors (Seriff & Durant, 1981), not assayable by steroid-binding methods. Conversely, 30 out of 50 ER+/PR− patients were post-menopausal (Figure 5). Here, one may wish to determine if such ER is non-functional (as aberrant receptor forms exist which bind ligand but are unable to activate transcription; Sherman *et al.*, 1978; Rusconi & Yamamoto, 1987), or whether it is still a biologically active ligand-dependent system which can be 'switched off' by classical anti-oestrogens such as tamoxifen. Clearly, the presence of both PR and pS2 is a strong indicator of endocrine responsiveness.

In conclusion, we have shown that, in breast tumour cytosols, a relationship between pS2 and total cathepsin D exists which could point to a possible role of pS2 in cathepsin D overexpression. Also, pS2 can be conveniently used in the determination of a functional ER.

We are most grateful to the Associazione Italiana per la Ricerca contro il Cancro (AIRC) for project grant support. We would like to thank the Gruppo di Studio per la Biologia del Carcinoma Mammario (GSBioCaM) for financial support.

### References

ALANKO, A., HEINONEN, E., SCHEININ, T., TOLPPANEN, E.S. & VIHKO, R. (1985). Significance of estrogen and progesterone receptors, disease-free interval, and site of first metastasis on survival of breast cancer patients. *Cancer*, **56**, 1696-1700.

ALEXIEVA-FIGUSCH, J., VAN PUTTEN, W.L.J., BLANKESTEIN, M.A., VAN DER WIJST, J.B. & KLIJN, J.G.M. (1988). The prognostic value and relationships of patient characteristics, estrogen and progestin receptors, and site of relapse in primary breast cancer. *Cancer*, **61**, 758-768.

ALLEGRA, J.C., LIPPMAN, M.E., THOMPSON, E.B., SIMON, R., BABLOCK, A., GREEN, L., HOFF, K.K., DO, H.M.T., IATKEN, S.C. & WARREN, R. (1980). Oestrogen receptor status: an important variable in predicting response to endocrine therapy in metastatic breast cancer. *Eur. J. Cancer*, **16**, 323-331.

BRADFORD, M.M. (1976). A rapid and sensitive method for the quantitation of microgram quantities of protein utilising the principle of protein-dye binding. *Anal. Biochem.*, **72**, 2428-254.

BROUILLET, J.P., THEILLET, C., MAUDELONDE, T., DEFRENNE, A., SIMONY-LAFONTAINE, J., SERTOUR, J., PUJOL, H., JEANTEUR, P. & ROCHEFORT, H. (1990). Cathepsin D assay in primary breast cancer and lymph nodes: relationship with c-myc, c-erb-B-2 and int-2 oncogene amplification and node invasiveness. *Eur. J. Cancer*, **26**, 437-441.

BYAR, D.P., SEARS, M.E. & MCGUIRE, W.L. (1979). Relationship between oestrogen receptor values and clinical data in predicting the response to endocrine therapy for patients with advanced breast cancer. *Eur. J. Cancer*, **15**, 299-310.

CAPPELLETTI, V., CORADINI, D., SCANZIANI, E., BENINI, E., SILVESTRINI, R. & DI FRONZO, G. (1992). Prognostic relevance of pS2 status in association with steroid receptor status and proliferative activity in node-negative breast cancer. *Eur. J. Cancer*, **28A**, 1315-1318.

CAVAILLES, V., GARCIA, M. & ROCHEFORT, H. (1989). Regulation of cathepsin D and pS2 gene expression by growth factors in MCF7 human breast cancer cells. *Mol. Endocrinol.*, **3**, 552-558.

CLARK, G.M., MCGUIRE, W.L., HUBAY, C.A., PEARSON, O.H. & MARSHALL, J.S. (1983). Progesterone receptors as a prognostic factor in stage II breast cancer. *N. Engl. J. Med.*, **309**, 1343-1347.

EORTC BREAST CANCER COOPERATIVE GROUP (1980). Revision of the standards for the assessment of hormone receptors in human breast cancers. *Eur. J. Cancer*, **16**, 1513-1515.

FISHER, B., WICKERHAM, D.L., BROWN, A. & REDMOND, C.K. (1983). Breast cancer estrogen and progesterone receptor values: their distribution, degree of concordance, and relation to number of positive axillary nodes. *J. Clin. Oncol.*, **1**, 349-358.

FOEKENS, J.A., RIO, M.C., SEGUIN, P., VAN PUTTEN, W.L.J., FAUQUE, J., NAP, M., KLIJN, G.M. & CHAMBON, P. (1990). Prediction of relapse and survival in breast cancer patients by pS2 protein status. *Cancer Res.*, **50**, 3832-3837.

GARCIA, M., CAPONY, F., DEROCQ, D., SIMON, D., PAU, B. & ROCHEFORT, H. (1985). Monoclonal antibodies to the oestrogen-regulated Mr 52,000 glycoprotein: characterization and immunodetection in MCF-7 cells. *Cancer Res.*, **45**, 709-716.

GOUSSARD, J., LECHEVREL, C., ROUSSEL, G., CREN, H., BERRA, O. & SALA, M. (1991) Immunoradiometric assay of pS2 protein in breast cancer cytosols. *Clin. Chem.*, **37**, 1759-1762.

HENRY, J.A., MCCARTHY, A.L., ANGUS, B., WESTLEY, B.R., MAY, F.E.B., NICHOLSON, S., CAIROS, J., HARRIS, A.L. & HORNE, C.H. (1990). Prognostic significance of the estrogen-regulated protein, cathepsin D, in breast cancer: an immunohistochemical study. *Cancer*, **65**, 265-271.

HENRY, J.A., PIGGOTT, N.H., MALLICK, U.K., NICHOLSON, S., FARNDON, J.R., WESTLEY, B.R. & MAY, F.E.B. (1991). pNR-2/pS2 immunohistochemical staining in breast cancer: correlation with prognostic factors and endocrine response. *Br. J. Cancer*, **63**, 615-622.

HORWITZ, K.B. & MCGUIRE, W.L. (1978). Estrogen control of progesterone receptors. *J. Biol. Chem.*, **72**, 2223-2228.

HORWITZ, K.B., MCGUIRE, W.L., PEARSON, O.H. & SEGALOFF, A. (1975). Predicting response to endocrine therapy in human breast cancer: a hypothesis. *Science*, **189**, 726-727.

JANICKE, F., SCHMITT, M., PACHE, L., ULM, K., HARBECK, N., HOFLER, H. & GRAEFF, H. (1993). Urokinase (uPA) and its inhibitor PAI-1 are strong and independent prognostic factors in node-negative breast cancer. *Breast Cancer Res. Treat.*, **24**, 219-226.

JOHNSON, M.D., TORRY, J.A., LIPPMAN, M.E. & DICKSON, R.B. (1993). The role of cathepsin D in the invasiveness of human breast cancer cells. *Cancer Res.*, **53**, 873-877.

KOERNER, F.C., GOLDBERG, D.E., EDGERTON, S.M. & SCHWARTZ, L.H. (1992). pS2 protein and steroid hormone receptors in invasive breast carcinomas. *Int. J. Cancer*, **52**, 183-188.

KUTE, T.E., SHAO, L.M., SUGG, K., LONG, R.T., RUSSELL, G.B. & CASE, L.D. (1992). Cathepsin D as a prognostic indicator for node-negative breast cancer patients using both immunoassays and enzymatic assays. *Cancer Res.*, **52**, 1-6.

MARSIGLIANTE, S., BISCOZZO, L., GRECO, S., LEO, G. & STORELLI, C. (1992). Relation of the cathepsin D level to the oestrogen receptor in human breast cancer. *Int. J. Clin. Lab. Res.*, **22**, 52-57.

MASIAKOWSKI, P., BREATHNACH, R., BLOCK, J., GANNON, F., KRUST, A. & CHAMBON, P. (1982). Cloning of cDNA sequences of hormone-regualted genes from the MCF-7 human breast cancer cell line. *Nucleic Acids Res.*, **10**, 7895-7903.

MATHIEU, M., ROCHEFORT, H., BARENTON, B., PREBOIS, C. & VIGNON, F. (1990). Interactions of cathepsin D and insulin-like growth factor-II on the IGF-II/mannose-6-phosphate receptor in human breast cancer cells and possible consequences on mitogenic activity of IGF-II. *Mol. Endocrinol.*, **4**, 1327-1335.

PARL, F.F., SCHMIDT, B.P., DUPONT, W.D. & WAGNER, R.K. (1984). Prognostic significance of estrogen receptor status in breast cancer in relation to tumor stage, axillary node metastasis, and histopathologic grading. *Cancer*, **54**, 2237-2242.

PREDINE, J., SPYRATOS, F., PRUD'HOMME, F., ANDRIEU, C., HACENE, K., BRUNET, M., PALLUD, C. & MILGROM, E. (1992). Enzyme-linked immunosorbent assay of pS2 in breast cancers, benign tumors, and normal breast tissues. *Cancer*, **69**, 2116-2123.

RAVDIN, P.M. (1993). Evaluation of cathepsin D as a prognostic factor in breast cancer. *Breast Cancer Res. Treat.*, **24**, 219-226.

RIO, M.C., BELLOCQ, J.P., GAIRARD, B., RASMUSSEN, U.B., KRUST, A., KOEHL, C., CALDEROLI, H., SCHIFF, V., RENAUD, R. & CHAMBON, P. (1987). Specific expression of the pS2 gene in subclasses of breast cancers in comparison with expression of the oestrogen and progesterone receptors and the oncogene ERBB2. *Proc. Natl Acad. Sci. USA*, **84**, 9243-9247.

RIO, M.C., BELLOCQ, J.P., DANIEL, J.Y., TOMASETTO, C., LATHE, R., CHENARD, M.P., BATZENSCHALAGER, A. & CHAMBON, P. (1988*a*). Breast cancer-associated pS2 protein: synthesis and secretion by normal stomach mucosa. *Science*, **24**, 705-708.

RIO, M.C., LEPAGE, P., DIEMUNSCH, P., ROITSCH, C. & CAMBON, P. (1988*b*). Structure primarie de la proéine humaine pS2. *C.R. Acad. Sci.*, **307**, 825-831.

ROCHEFORT, H. (1992). Cathepsin D in breast cancer: a tissue marker associated with metastasis. *Eur. J. Cancer*, **28A**, 1780-1783.

ROCHEFORT, H., CAPONY, F., GARCIA, M., CAVAILLES, V., FREISS, G., CHAMBON, M., MORISSET, M. & VIGNON, F. (1987). Estrogen-induced lysosomal proteases secreted by breast cancer cells: a role in carcinogenesis? *J. Cell. Biochem.*, **35**, 17-29.

RUSCONI, S. & YAMAMOTO, K.R. (1987). Functional dissection of the hormone binding and DNA binidng activities of the glucocorticoid receptor. *EMBO J.*, **6**, 1309-1315.

SCHWARTZ, L.H., KOERNER, F.C., EDGERTON, S.M., SAWICKA, J.M., RIO, M., BELLOCQ, J., CHAMBON, P. & THOR, A.D. (1991). pS2 expression and response to hormonal therapy in patients with advanced breast cancer. *Cancer Res.*, **51**, 624-628.

SERRIF, A.M. & DURANT, J.R. (1981) Evidence that estrogen receptor negative/progesterone receptor positive breast and ovarian carcinomas contain estrogen receptor. *Cancer*, **48**, 1215-1220.

SHERMAN, M.R., PICKERING, L.A., ROLLWAGEN, F.M. & MILLER, L.K. (1978). Mero-receptors: proteolytic fragments of receptors containing the steroid binding site. *Fed. Proc.*, **37**, 23-30.

SPYRATOS, F., BROILLET, J.P., DEFRENNE, A., HACENE, K., ROUESSE, J, MAUDELONDE, T., BRUNET, M., ANDRIEU, C., DESPLACES, A. & ROCHEFORT, H. (1989). Cathepsin D: an independent prognostic factor for metastasis of breast cancer. *Lancet*, **8672**, 1115-1118.

TANDON, A.K., CLARK, G.M., CHAMNESS, G.C., CHIRGWIN, J.M. & MCGUIRE, W.L. (1990) Cathepsin D and prognosis in breast cancer. *N. Engl. J. Med.*, **322**, 297-302.

THORPE, S.M., ROCHEFORT, H., GARCIA, M., FREISS, G., CHRISTENSEN, I.J., KHALAF, S., PAOLUCCI, F., PAU, B., RASMUSSEN, B.B. & ROSE, C. (1989). Association between high concentrations of Mr 52,000 cathepsin D and poor prognosis in primary breast cancer. *Cancer Res.*, **49**, 6008-6014.

WESTLEY, B. & ROCHEFORT, H. (1980). A secreted glycoprotein induced by oestrogen in human breast cancer cell lines. *Cell*, **20**, 353-362.